# An open source microcontroller based flume for evaluating swimming performance of larval, juvenile, and adult zebrafish

**DOI:** 10.1371/journal.pone.0199712

**Published:** 2018-06-26

**Authors:** Jeffrey J. Widrick, Devin E. Gibbs, Benjamin Sanchez, Vandana A. Gupta, Anna Pakula, Christian Lawrence, Alan H. Beggs, Louis M. Kunkel

**Affiliations:** 1 Division of Genetics and Genomics, The Manton Center for Orphan Disease Research, Boston Children’s Hospital and Harvard Medical School, Boston, MA, United States of America; 2 Department of Neurology, Division of Neuromuscular Diseases, Beth Israel Deaconess Medical Center, Boston, MA, United States of America; 3 Department of Medicine, Division of Genetics, Brigham and Women’s Hospital, Boston, MA, United States of America; 4 Aquatic Resources Program, Boston Children’s Hospital, Boston, MA, United States of America; National Institutes of Health, UNITED STATES

## Abstract

Zebrafish are a preferred vertebrate model for delineating genotype-phenotype relationships. One of the most studied features of zebrafish is their exceptional swimming ability. By 7 days postfertilization (dpf), zebrafish spend over two-thirds of their time engaged in spontaneous swimming activity and several months later they are capable of attaining some of the fastest swimming velocities relative to body length ever recorded in the laboratory. However, laboratory-assembled flumes capable of achieving the slow flow velocities characteristics of larvae as well as the relatively fast maximal velocities of adults have not been described in sufficient detail to allow easy replication. Here we describe an easily assembled, open-source zebrafish-scaled flume for assessing swimming performance. The flume uses two independent spherical-impeller pumps modulated by a microcontroller to achieve flow velocities ranging from 1 to 70 cm s^−1^. The microcontroller also monitors water temperature and flow velocity and sends these data to a personal computer for real-time display and storage. Incremental protocols for assessing maximal swimming speed (*U*_*max*_) were developed, stored in custom software, and then uploaded to the microcontroller in order to assess performance of larval (14, 21, 28 dpf), juvenile (35, 42 dpf), and adult (8, 22 month) zebrafish. The flume had sufficient range and sensitivity to detect developmental changes in *U*_*max*_ of larvae and juveniles, an 18–24% faster *U*_*max*_ of adult males vs. females, and a 14–20% age-related reduction in *U*_*max*_ for the oldest zebrafish. Detailed information is provided to assemble and operate this low-cost, versatile, and reliable tool for assessing zebrafish swimming performance.

## Introduction

Zebrafish (*Danio rerio*) are small freshwater fish that are used extensively for investigations of vertebrate phenotype-genotype relationships. One of the most recognizable phenotypic characteristics of zebrafish is their robust swimming activity. Zebrafish display brief, intermittent bursts of high-speed propulsion as soon as they emerge from the chorion 2–3 days post fertilization (dpf) [1, 2]. By 5 dpf, the posterior chamber of the swim bladder has inflated [3], larvae achieve neutral buoyancy [4], and swimming takes on an intermittent beat and glide pattern [1]. During these early developmental stages, motility can be reliability evaluated using touch-evoked escape responses [2, 5]. However, it is difficult to use touch-evoked responses beyond 7 dpf [6] because larvae now spend about two-thirds of their time engaged in spontaneous swimming activity [7].

An alternative method of studying swimming performance is to use a flume or swim tunnel to manipulate swimming velocity [8]. To obtain maximal swimming speed, flow can be systematically increased in discrete stages until the fish can no longer overcome the current and continue swimming. If the protocol uses relatively long duration stages so that the entire test requires from one to several hours to complete, the water velocity at exhaustion is termed the critical swimming speed, or *U*_*crit*_. If the swimming stages are of shorter duration, so that the test is completed in minutes instead of hours, the maximal swimming speed is termed *U*_*burst*_ or *U*_*max*_. It has been proposed that *U*_*crit*_ is limited by oxygen and substrate deliver while *U*_*max*_ is determined primarily by the mechanical properties of the active musculature [9].

In zebrafish, *U*_*crit*_ rises from ≈ 3 cm s^−1^ in 5 dpf larvae to 40 − 55 cm s^−1^ in adults [10–13]. *U*_*max*_ exceeds *U*_*crit*_ by about 15% when both are evaluated in the same group of adult zebrafish [11]. Taken together, these results indicate that adult zebrafish are capable of attaining sustained swimming speeds that allow them to cover > 15 times their body length every second. This makes zebrafish one of the fastest fish species per body size ever studied in the laboratory [10].

The small size and relatively slow swimming speeds of larval zebrafish, the large changes in absolute swimming speed that occur during development and maturation, and the fast peak swimming velocities of adults place unique demands on flume design. Commercially-available flumes, based on designs originally described by Brett [14] and Blažka et al. [15], are usually scaled for studying fish in the g to kg range [8]. The smallest of these flumes may have peak flow velocities that are at the lower end of adult zebrafish *U*_*crit*_ and *U*_*max*_. For these reasons, investigators often fabricate custom flumes for their zebrafish studies [10, 12, 16]. However, the materials, construction, and performance of these laboratory-constructed instruments are usually not reported in sufficient detail to enable easy or accurate replication.

Here we describe the design, calibration, and operation of a microcontroller based, laboratory assembled flume specifically scaled for zebrafish. The microcontroller is built on an open-source platform that is configured using a program developed in our laboratory. This enables users to create, store, and re-administer custom test protocols with high reliability. Most of the remaining flume components can be used off-the-shelf with only minimal modification. To demonstrate the range of flow velocities achieved by the flume, we developed age-appropriate protocols and successfully measured *U*_*max*_ across the zebrafish lifespan, from 14 dpf larvae, through juveniles, adults, and into senescence. This tool and the supporting protocols may have applications in zebrafish studies in which quantification of swimming behavior is an important phenotypic variable, such as characterization of different zebrafish mutant lines, disease models, and therapeutic interventions.

## Materials and methods

We designed a small, portable flume that was operated by a microcontroller. Here, we provide an overview of its assembly, calibration, and use. The reader is referred to the Supporting Information for further details regarding materials, construction, software, calibration, and operation.

### Flume design

**Fig 1:**
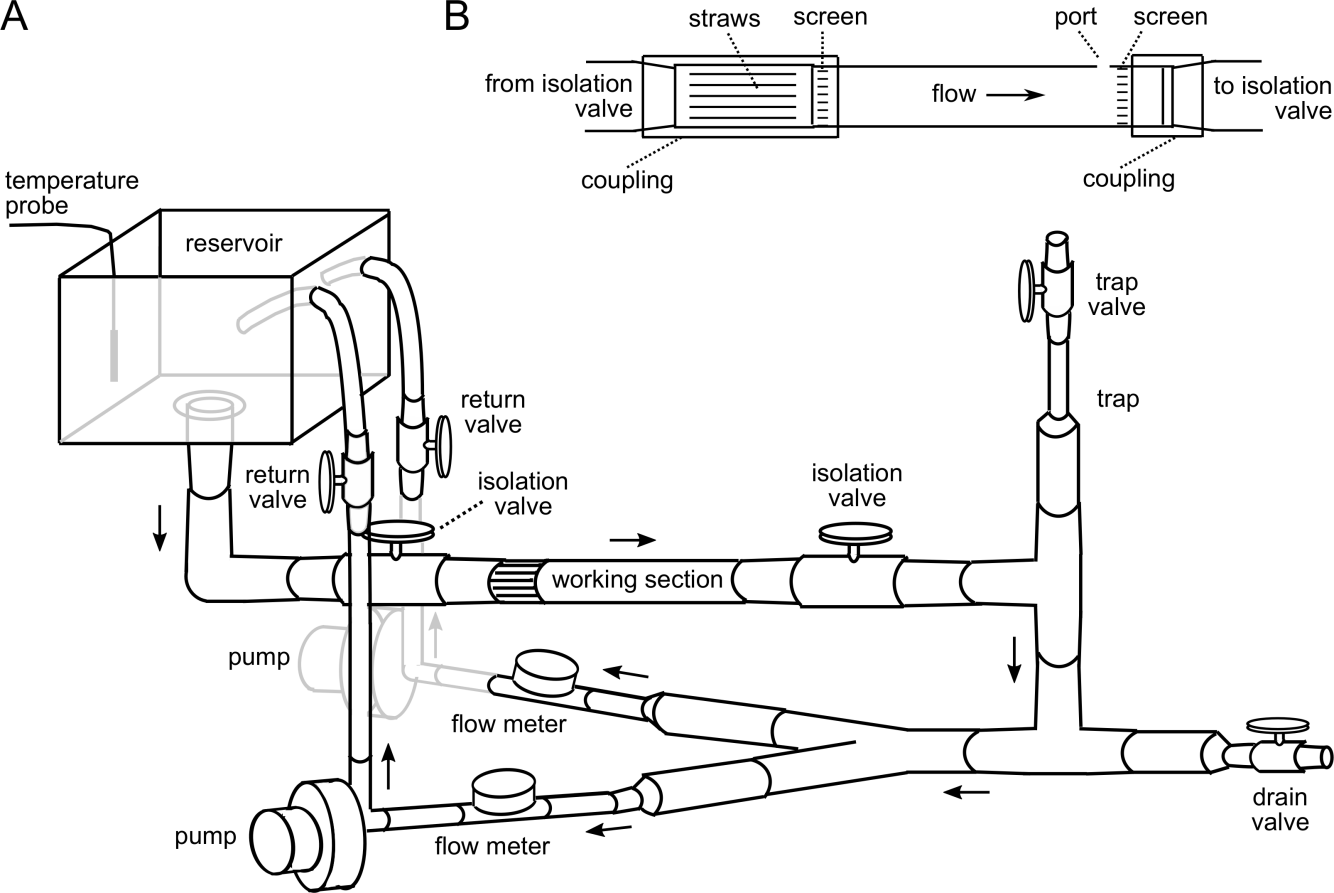
Design of the flume. (A) Schematic diagram of the flume. (B) Expanded view of the working section. Diagrams are not drawn to scale. See S1 Appendix for details on components and construction.

The overall design of the flume is illustrated in Fig 1A. Briefly, water exited an elevated reservoir into the working section of the flume. Upon leaving the working section, water was diverted into one of two similar circuits, each containing an in-line flow meter and a magnetically driven spherical impeller pump. Water was pulled through the flume by the pumps and expelled back up into the elevated reservoir.

The working section of the flume was made from a clear polycarbonate cylindrical tube with an interior cross-sectional area (CSA) of 5.07 cm^2^ (Fig 1B). Stainless steel screens placed across the entry and exit points of the working section confined the fish to a 12.5 cm long section of the tube. We choose these dimensions for the working section because they are similar to the dimensions of one of the smallest commercially available flumes (Loligo Systems, model SW10000). This commercial flume is scaled for 1–4 g fish. Because adult zebrafish rarely exceed 0.5 g in mass, our working section dimensions are more than adequate.

Immediately prior to the upstream screen, water passed through a 4 cm long, tightly packed array of plastic straws to ensure that flow was laminar. A small access port was drilled through one wall of the working section near its downstream end. This port was useful for loading and removing larvae and juveniles from the working section and for purging the working section of air bubbles. During flume operation, the access port was sealed with a small piece of Parafilm. A vertical column downstream from the working section served as a bubble trap. The column could be purged by opening the vent valve.

A pair of valves were positioned immediately down- and up-stream from the working section. These valves allowed the user to isolate the working section so it could be accessed for adding or removing fish. Valves were also located between the pumps and their outflow into the reservoir. These valves could be closed to prevent back-flow through the circuit when an individual pump was powered down. They could also be adjusted to reduce flow through the pumps in order to provide slower flow through the working section when studying larvae and juveniles. The temperature of the flume water was controlled by circulating warm water from a thermostatted water bath through several coils of polyvinyl chloride (PVC) tubing that were submerged in the reservoir.

### Microprocessor

**Fig 2:**
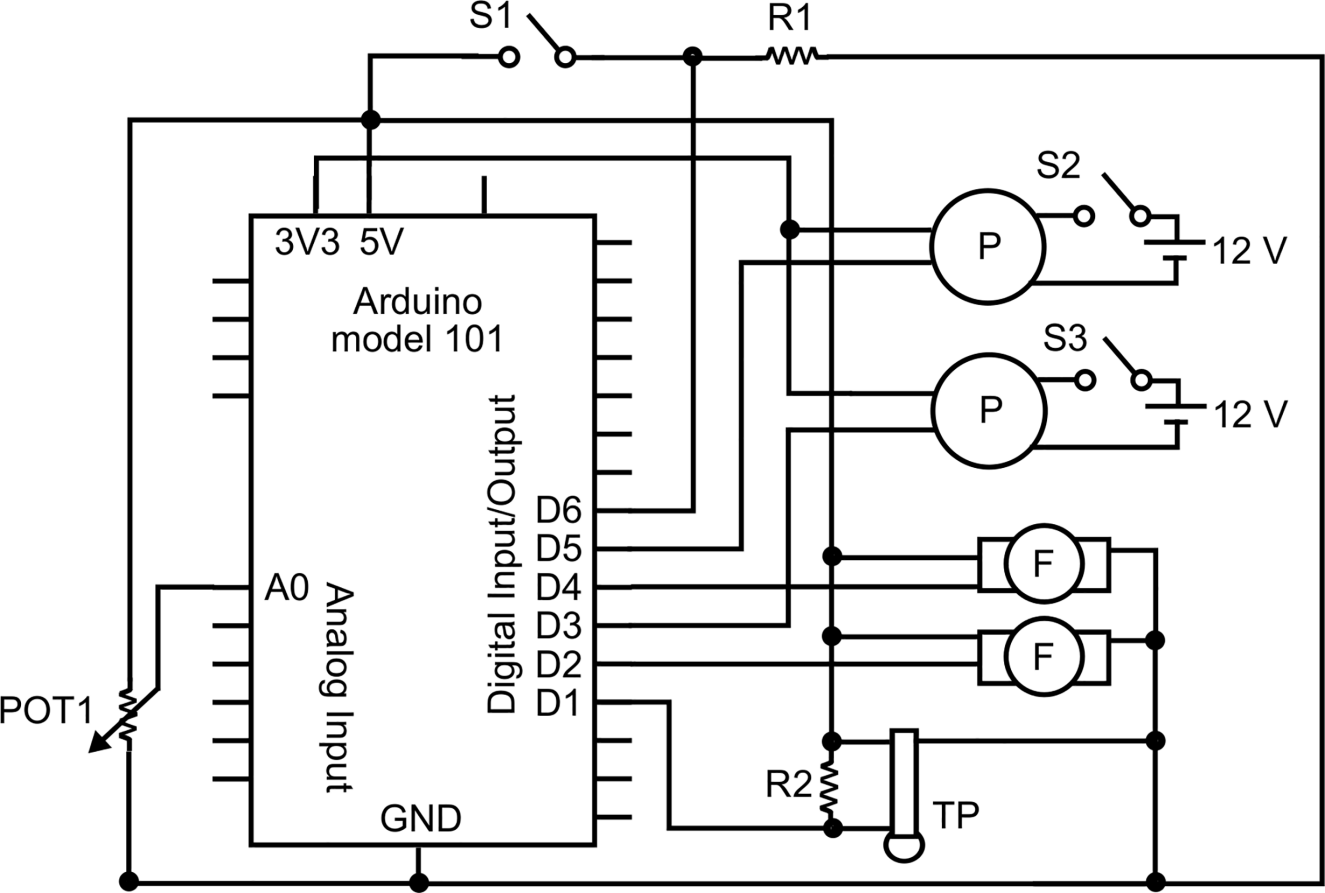
Arduino wiring. Abbreviations: R1, 10 kΩ; S1, switch (for exiting current mode of operation); POT1, potentiometer (for manual control of flow); R2, 4.7 kΩ; S2 and S3, switches (for powering pumps); GND, ground; 3V3, 3.3 V output; P, pump; F flow meter; TP, temperature probe. See S2 Appendix for details on components.

An Arduino model 101 open source microcontroller platform, which uses an Intel Curie module, was used to control and monitor the flume. As shown in Fig 2, the pumps were controlled by two of the pulse width modulation (PWM) output pins of the Arduino. The PWM value, which varied from 0 to 255, was inversely related to the pump output. Each pump had its own PWM signal and power supply so that they could be controlled independently.

### Software

The Arduino Independent Development Environment (IDE) was used to create a program, FlowControl, for calibrating and operating the flume. The FlowControl code and a users manual have been deposited on GitHub (https://github.com/jjwidrick/flume-project). FlowControl was uploaded from a personal computer (PC) to the Arduino via a standard USB A-B printer cable. The PC was required to run the flume because the microprocessor, 1) drew its power via the USB cable connection, and 2) sent data to the serial monitor of the IDE for display on the PC’s monitor.

FlowControl had three modes of operation. Mode 1 allowed the user to run a custom protocol that had been previously entered into the microcontroller source code. Mode 2 enabled the investigator to control the flow rate through manual adjustment of the potentiometer (POT1 in Fig 2). Mode 3 was used to automatically obtain the data required for the PWM signal vs. flow rate calibration (detailed below). Finally, the flume had a manual shut-off switch that was monitored by the microprocessor. Regardless of the operating mode, when the polarity of this switch changed, the PWM signal was set to 255 for the next data acquisition cycle. This shut off the pumps and stopped flow through the flume.

The following data acquisition cycle was repeated continuously regardless of the mode of operation. The microcontroller counted pulses from each flow meter for 4 s. The microprocessor calculated the flow rate, read a temperature probe inserted into the reservoir and calculated water temperature, and then transferred these data, along with other pertinent information including time stamps, to the serial monitor of the IDE running on the PC. Data packages were updated every 5 s until a mode timed out or the user manually terminated operation. Additional details regarding the FlowControl software and a sample of the data sent to the serial monitor are presented in S3 and S4 Appendixes, respectively. At the end of a trial, data could be copied from the serial monitor onto the PC’s clipboard, pasted into a text editor, and saved to disk for further analysis.

### Flow meter calibration

The relationship between flow meter pulse frequency output and flow rate was obtained by collecting and measuring the volume of water output by the pump for a specific period of time while simultaneously monitoring the flow meter pulse frequency (see S3 Appendix for details). The relationship between the flow meter pulse frequency and the observed flow rate was used to determine calibration constants for each meter.

### PWM versus flow calibration

The pumps were driven through a series of 10 PWM values that encompassed slow to fast flow rates. At each PWM level, data were collected for 30 s. Flow rate was averaged over the final 15 s of each stage. The relationship between mean flow rate and the PWM value was described using a 2^nd^ order polynomial regression. The coefficients from this polynomial were entered into the source code so that for any target flow rate, the software could calculate an associated PWM value. This calibration was performed at the start of each data collection period or whenever the flume was altered in a way that would influence the resistance to flow.

### Flow calculations

The volume of water passing through a flow meter per unit time (in ml s^−1^) was calculated as (*p/s*×*m*)+*x*, where *p* is the number of pulses output by the flow meter, *s* is the number of seconds that pulses were counted, and *m* and *x* are the meter’s slope and y-intercept calibration coefficients, in ml pulse^−1^ and ml, respectively. Flow through the working section (in cm s^−1^) was calculated as (*F*_1_ + *F*_2_)*/*CSA, where *F*_1_ and *F*_2_ are the flows rates through flow meters 1 and 2, respectively, and CSA is the cross-sectional area of the working section of the flume (in cm^2^).

### Modifications for studying larvae and juveniles

The pumps would stall before reaching the slowest target velocities required for studying larvae and juveniles. To achieve slow flow rates, pump 2 was powered down and its outlet valve was closed. Pump 1 remained operative with its outlet valve partially closed. This restricted the pump output thereby reducing the rate that water was drawn through the flume. The PWM versus flow calibration was then performed as described above. The outlet valve remained locked in position for subsequent data collection.

### Zebrafish

To test the utility of the flume, we measured swimming performance of zebrafish across their lifespan, including larval, juvenile, and adult fish. Zebrafish (*Danio rerio*, AB strain) were raised and maintained using standard procedures. Details of husbandry and environmental conditions are available on protocols.io (DOI: dx.doi.org/10.17504/protocols.io.mrjc54n). The study was carried out in strict accordance with all federal regulations and guidelines regarding the humane care and use of laboratory animals. The Institutional Animal Care and Use Committee at Children’s Hospital Boston approved all experiments in which animals were used (IACUC protocol # 15-09-2989R).

### Measurement of *U*_max_

Maximal swimming speed, *U*_*max*_, was determined for individual zebrafish using a protocol in which flow velocity was systematically incremented in stages until the fish was exhausted. A fish was considered exhausted when it was swept against the downstream screen and would not resume swimming despite several mechanical taps to the working section.

After the protocol was completed, the fish was removed from the flume. Larvae and juveniles were euthanized with tricaine (MS-222) following standard procedure (< 15 dpf, 30 mg/100 mL fish water; > 15 dpf, 1 g/100 mL fish water). Adults were lightly anesthetized with 0.01% tricaine. Standard length (SL), defined as the distance from the tip of the snout to the intersection of the axial musculature with the caudal tail, was measured with a micrometer. Adults were then quickly returned to their tanks where they were periodically observed until they recovered from anesthesia.

### Data analysis

*U*_*max*_ was expressed relative to SL in order to allow comparisons between fish of different sizes and ages. R statistical software [17] and the following base functions and packages were used for statistical analysis: the lm function for linear and polynomial regression, the segmented package [18] for piecewise regression, the aov function for two-way ANOVA. Results are expressed as mean ± standard error (SE).

## Results

### Flow meter calibration

**Fig 3:**
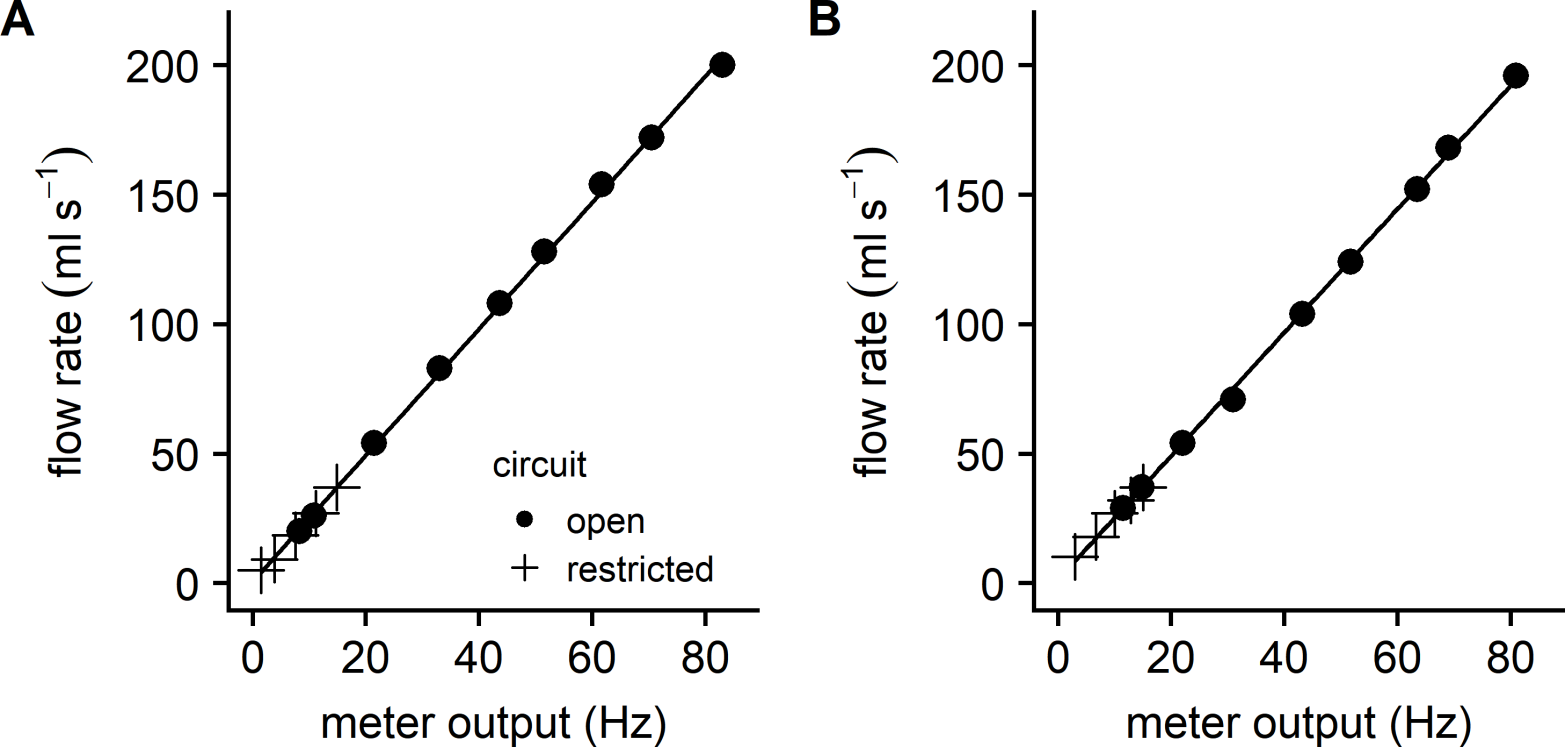
Calibration of the flow meters. Linear regression between flow meter pulses per s and the measured flow rate. The filled circles represent data collected with no circuit restriction in order to maximize the range and magnitude of the flow rate for studies of adult fish. The cross symbols represent data collected with the circuit partially restricted in order to slow the flow rate into a range suitable for studying larvae and juveniles. (A) Regression results for meter 1: *y* = 2.44*x* + 0.79, *R*^2^ = 0.999. (B) Regression results for meter 2: *y* = 2.38*x* + 1.55, *R*^2^ = 0.999.

The flow meters used the Hall effect to convert each rotation of an internal turbine into a change in voltage. The microcontroller processed this intermittent voltage signal into a pulse frequency which was then used to calculate the flow rate. Fig 3 illustrates the relationship between the flow meter pulse frequency and the volume of water flowing through each of the flow meters. The relationship was linear over the range of flow rates expected for larval through adult zebrafish. The two flow meters had very similar slopes and y-intercepts.

As a further check on flow characteristics, we injected methylene blue into the flume just upstream from the flow straighteners and filmed the movement of the dye as it flowed through the working section. Video analysis was used to determine the rate that dye flowed through the flume. Flow velocities derived from dye movement and from the flow meters were in good agreement over the range of flow velocities used in this study. In addition, we observed minimal turbulence as the dye was drawn through the working section, suggesting that flow was laminar.

### PWM versus flow calibration

**Fig 4:**
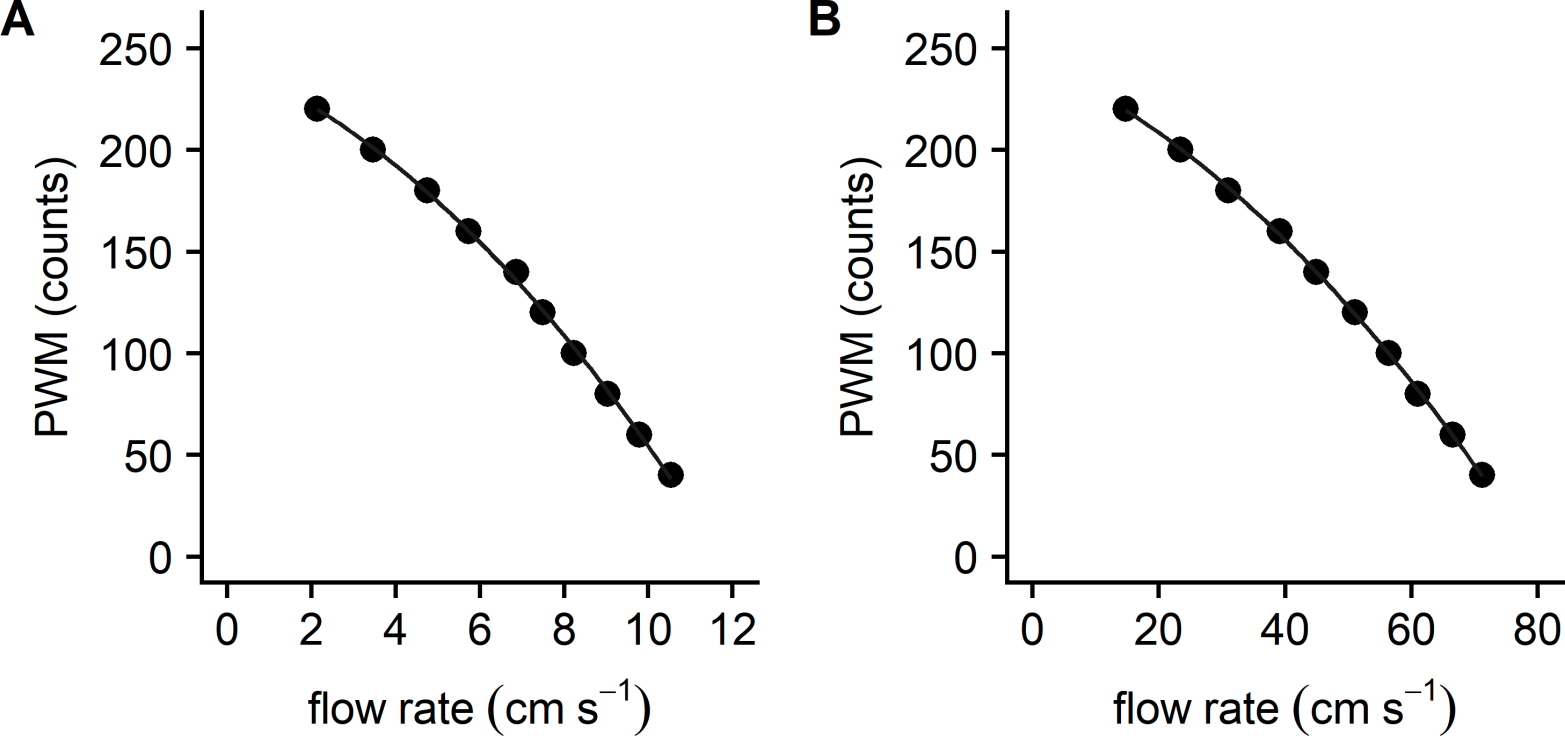
The relationship between flow rate and the PWM signal. (A) Slow flow rates evaluated with restricted flow through the flume circuit. Polynomial regression results: *y* = −8.430*x* − 1.042*x*^2^ + 242.8, *R*^2^ = 0.999. (B) Fast flow rates evaluated with no restriction to flow. Polynomial regression results: *y* = −1.374*x* − 0.021*x*^2^ + 244.4, *R*^2^ = 1.00.

When running a predetermined protocol, the flow through the flume was dependent on pump output which in turn was proportional to the PWM signal from the microcontroller. In order to accurately reproduce a user-defined target flow rate, the relationship between the PWM signal and the measured flow had to be established before each test session. Fig 4 shows that the relationship between the PWM value under both slow-flow (panel A: velocities appropriate for larvae and juveniles) and fast-flow (panel B: velocities appropriate for adults) test conditions could be well described by polynomial functions.

### Reproducibility

**Fig 5:**
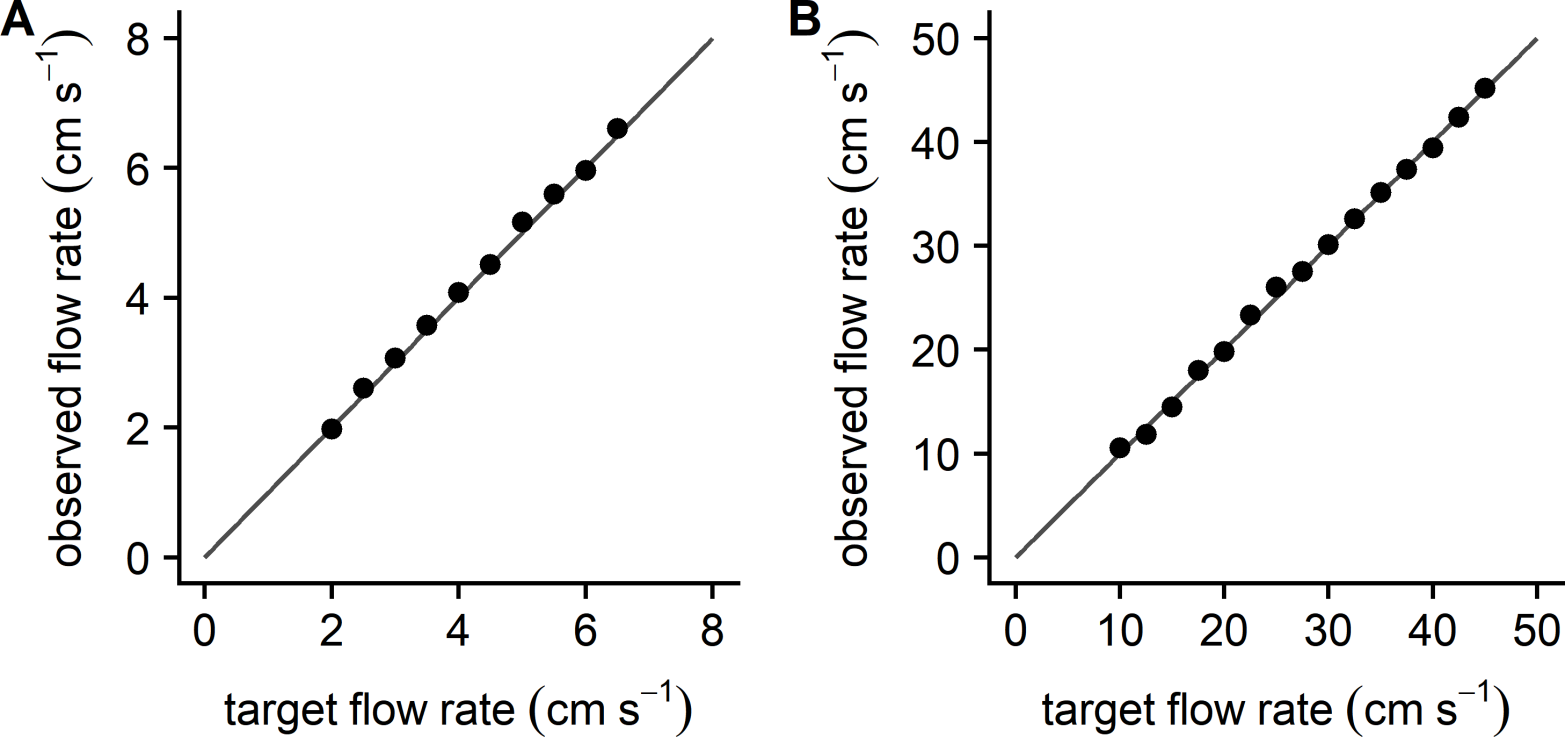
Relationship between target and observed flow rates. Data points represent the average observed flow rate (between 5–30 s of the trial) versus the target or expected flow rate at each stage of a slow flow protocol (A) and a fast flow protocol (B). The solid line is the line of identity. Mean deviation (average of the differences between observed and target flow rates across entire trial) was 0.067 cm s^−1^ for the slow flow protocol and 0.083cms^−1^ for the fast flow protocol.

Following the calibration of PWM signal, we could program the microcontroller to maintain a target flow rate for a specific period of time (see S3 Appendix for details). This allowed us to create and store customized protocols for assessing zebrafish performance. Fig 5 illustrates two representative trials, one conducted on a 14 dpf larvae, i.e. at the slowest flow rates, and one conducted on an 8 month adult zebrafish, i.e. using the fastest flows. These figures illustrate the relationship between the programmed target flow rates and the corresponding flow through the working section. The observed flow rates tracked the linear rise in target flow rates across the full range of flow velocities. To quantify this relationship, we calculated a mean deviation as the average difference between the observed and target flow across all stages of a *U*_*max*_ trial. The mean deviation from the target flow for all *U*_*max*_ evaluations in this study (n = 53) was 0.12 ± 0.06 cm s^−1^.

### U_max_ of larval, juvenile, and adult zebrafish

To evaluate the performance of the flume under actual laboratory conditions, we created incremental swimming protocols for quantifying *U*_*max*_ of larval, juvenile, and adult zebrafish. Our adult protocol was a modification of a protocol previously used for zebrafish of similar age [11]. Protocols for larval and juvenile fish were then based on the adult protocol but with initial flow velocities and velocity increments adjusted based on preliminary experiments. The initial flow velocities, the velocity increments, the duration of each stage, and the average time required for protocol completion are summarized in Table 1.

**Table 1:** Protocols for evaluating maximal swimming speed (*U*_*max*_) of each group of zebrafish.

group	initial velocity	velocity increment	stage duration	total duration
	(cm s^−1^)	(cm s^−1^)	(s)	(min:s)
larvae	2	0.5	30	4:30
juvenile	6	1.5	30	6:45
adult	10	2.5	30	6:30

Total duration is the average evaluation time for the *U*_*max*_ trials in the present study.

**Fig 6:**
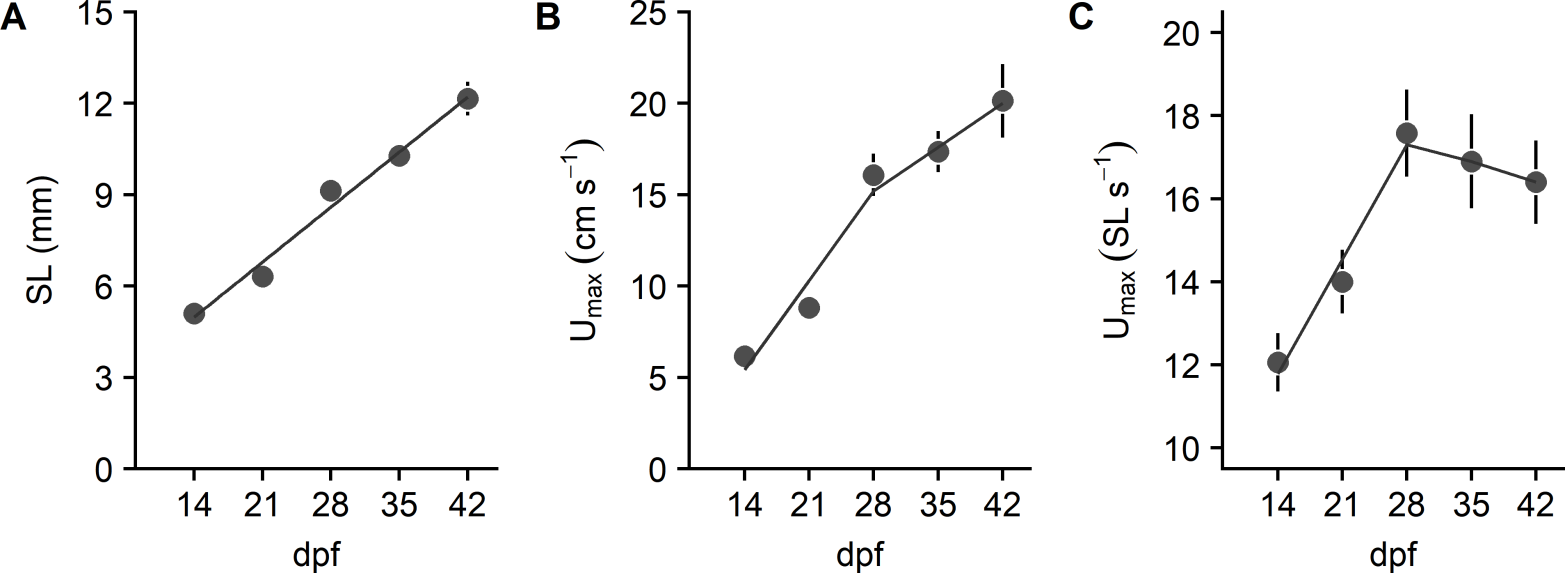
Standard length and maximal swimming speed (*U*_*max*_) of larval and juvenile zebrafish. (A) Standard length (*SL*). The relationship between SL and age (dpf) was described by *y* = 0.258*x* + 1.377, *R*^2^ = 0.920. (B) Absolute *U*_*max*_. The slope of the relationship between *U*_*max*_ in cm s^−1^ and age (dpf) for 14–28 dpf fish was 0.698, with 95% confidence intervals of 0.414 to 0.981. The slope of the relationship for 28–42 dpf fish was 0.344, with 95% confidence intervals of -0.173 to 0.862. (C) *U*_*max*_ relative to standard length. The slope of the relationship between *U*_*max*_ in cm s^−1^ and age (dpf) for 14–28 dpf fish was 0.394, with 95% confidence intervals of 0.4186 to 0.603. The slope of the relationship for 28–42 dpf fish was -0.071, with 95% confidence intervals of -0.452 to 0.310. Values are mean ± SE for 5, 5, 5, 6, and 6 fish at 14, 21, 28, 35, and 42 dpf, respectively.

Larvae and juvenile fish (N = 27) showed a linear rise in SL from 14 to 42 dpf (Fig 6A). Absolute *U*_*max*_ also rose across this same time period but appeared to show a breakpoint between 21 and 28 dpf (Fig 6B). Therefore, we used piecewise or segmented regression to fit the initial three time points and the final three time points [18]. Results indicated that *U*_*max*_ rose at a two-fold greater rate from 14–28 dpf than it did from 28–42 days. This breakpoint in swimming speed was even more evident when we examined *U*_*max*_ relative to SL (Fig 6C). Relative *U*_*max*_ rose until 28 dpf and then tended to decline slightly through 42 dpf. Because the 95% confidence intervals include 0, the slope is not significantly different from horizontal. Thus, *U*_*max*_ rose out of proportion to growth until 28 dpf. After 28 dpf, *U*_*max*_ changed in proportion to growth.

**Fig 7:**
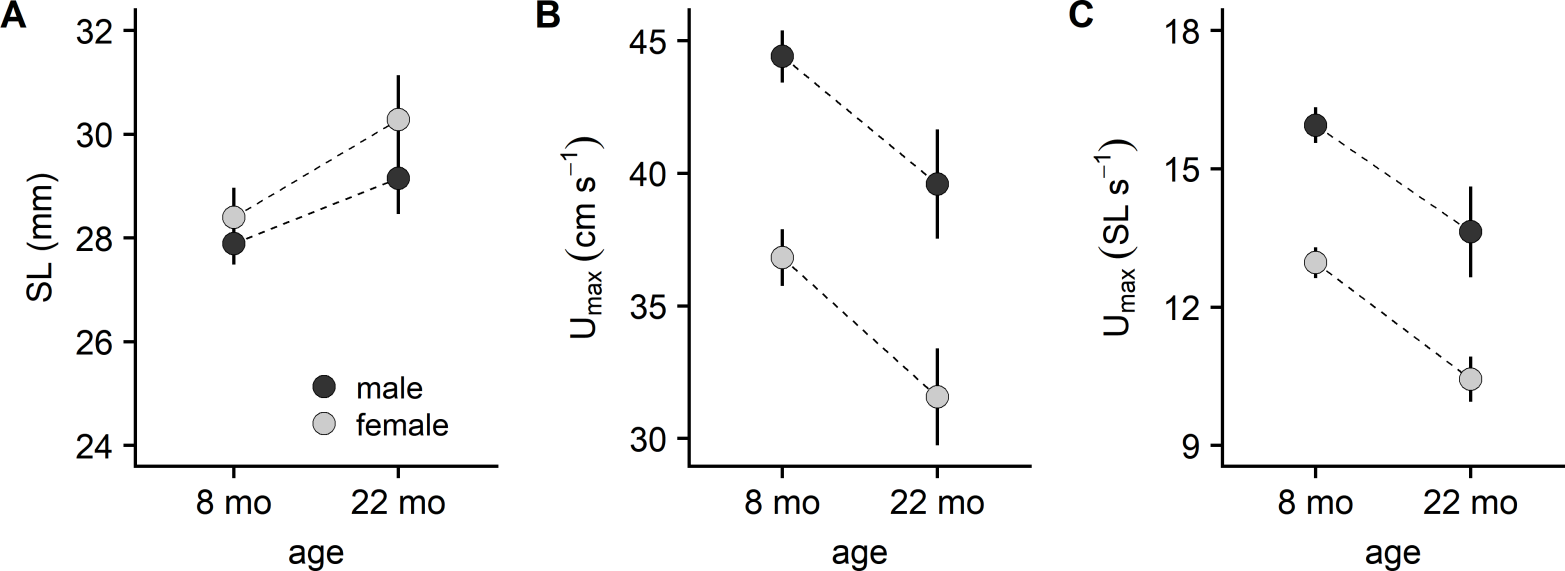
Maximal swimming speed (*U*_*max*_) of adult zebrafish. Adults were classified by sex and age and analyzed using two-way ANOVA. (A) Absolute *U*_*max*_. There was a significant effect of age (*P* = 0.0019), a significant effect of sex (*P <* 0.0001), but no significant age by sex interaction (*P* = 0.8798). (B) *U*_*max*_ relative to standard length (*SL*). There was a significant age effect (*P* = 0.0002), a significant sex effect (*P <* 0.0001), but no significant age by sex interaction (*P* = 0.8416). Values are mean ± SE for 9, 8, 3, and 3 eight month males, eight month females, twenty-two month males, and twenty-two month females, respectively.

Adult zebrafish were classified as male or female and as young (8 month; 9 males, 8 females) or old (22 month; 3 males, 3 females). The old fish were slightly longer in length than the young fish (Fig 7A) but this had little effect on performance as the results that follow held for both absolute (Fig 7B) and relative (Fig 7C) *U*_*max*_. Sex was found to have a significant impact on *U*_*max*_, regardless of the age of the fish. On average, the *U*_*max*_ of males was about 8 cm s^−1^, or 3 SL s^−1^, faster than that observed for females. Age also impacted *U*_*max*_. The *U*_*max*_ of 22 month old fish was roughly 5 cm s^−1^, or 2.5 SL s^−1^, slower than 8 month old fish. This age-related effect on *U*_*max*_ was similar for male and female fish.

## Discussion

We designed a swim flume for investigators who wish to study locomotion of small laboratory fish. While we designed the flume specifically for zebrafish, it should also be applicable for other small laboratory fish models, such as guppies (*Poecilia reticulata*) and madaka (*Oryzias latipes*).

The flume has a number of features that make it an attractive alternative to commercial and other laboratory-assembled flumes. First, the flume is small in scale but capable of attaining the full range of flow velocities one would expect to encounter when studying zebrafish. This makes it particularly applicable for investigators studying developmental and age-related changes across the zebrafish lifespan. Second, flow is modulated by a microcontroller. This enables investigators to control flow either manually or programmatically. The latter approach allows the user to define, store, and recall custom swim protocols, eliminating the potential for trial-to-trial variation when flow and timing are controlled manually. The use of this open-source hardware platform and soft-ware confers other advantages, including low-cost, the ability to more easily incorporate custom design parameters, and access to an extensive on-line community of expertise [19]. Third, the microcontroller collects relevant data during flume operation and processes this information to yield continuous measures of flow velocity and water temperature. At the conclusion of a protocol, these data can be easily copied and saved into the PC as text files. The use of text files eliminates proprietary data formats and ensures access to the data on any computer. Fourth, the flume is built with off-the-shelf components that require only minor modification, making materials and construction accessible to a wide range of potential users. Finally, the flume has a relatively small footprint and requires no special plumbing. This makes the flume portable for use in a fish facility, at the lab bench, or even in the field.

As a practical test of our flume and protocols, we measured *U*_*max*_ of larval, juvenile, young adult, and old adult zebrafish. Some of these developmental stages have been studied previously (Table 2). Note that our protocols were considerably shorter in duration than those compiled in Table 2 but our maximal swimming velocities for both 14 dpf larvae and for adults are in good agreement with literature values. Although our stages were shorter in duration, our velocity increments (0.50 cm s^−1^ for 14 dpf larvae; 2.5 cm s^−1^ for adults) were smaller than the increments (0.64 cm s^−1^ for 14 dpf larvae; 4 − 6 cm s^−1^ for adults) used in the other studies compiled in Table 2.

**Table 2:** Summary of literature values for maximal swimming speed (*U*_*max*_) of zebrafish.

	temperature	duration	14 dpf	adult	old
	(°C)	(min)			
Plaut [10]	28	65		15.5	
Fiaz et al. [12]	28	99	10.3		
Seebacher and Walter [16]	25	17		13.7	
Gilbert et al. [11]	28	79		14.4 *m* = *f*	
	28	56			9.5 *m* = *f*
		9		16.3 *m* = *f*	
		7			11.2 *m* = *f*
Conradsen et al. [13]	28	54		14.8 ^*f*^	
		63		17.9 ^*m*^	
present study	25	4	12.1		
		6		13.0 ^*f*^	
		5			10.4 ^*f*^
		7		15.9 ^*m*^	
		6			13.6 ^*m*^

All maximal swimming speeds expressed as mean SL s−^1^, where SL is standard length. Values were taken directly from Plaut [10] and Seebacher and Walter [16]. For Gilbert et al. [11], values were calculated by dividing the reported mean maximal swimming speed in cm s−^1^ by the reported mean SL. For Fiaz et al. [12], mean maximal swimming speed in cm s−^1^ and mean SL were estimated from figures and used to calculate maximal swimming speed in SL s−^1^. For Conradsen et al. [13] maximal swimming speed means were calculated from the first trial of their supplemental data. Adults were 3.5 − 12 months old depending on the study, and old fish were 25 − 30 months in Gilbert et al. [11] and 22 months in the present study. Duration column indicates the average time required for evaluation, calculated from the initial swimming speed, the average maximal swimming speed, the speed increment per stage, the average number of stages, and the duration of each stage. Sex distributions indicated by superscripts: ^*m* = *f*^, equal number of males and females; ^*m*^, males only; ^*f*^, females only; no superscript, no sex distribution reported.

Smaller velocity steps between stages may have improved our ability to resolve developmental changes in larvae and juvenile fish. For instance, we distinguished two phases in the early maturation of *U*_*max*_: an initial rapid rise in absolute *U*_*max*_ followed at the 28 dpf time point by a considerably slower change. During this initial rapid phase, *U*_*max*_ increased out of proportion to the linear growth in larval SL whereas later in development *U*_*max*_ and growth appeared to occur in parallel. There are many developmental changes in physiology and biomechanics that occur when fish transition from larvae to juveniles around 28 dpf. These include maturation of the swimbladder [4], squamation and fin development [20], appearance of adult muscle fiber types [21], morphological changes affecting fluid dynamics [22], and functional gill development [23]. However, not all of these changes are immediately beneficial to swimming performance [22], indicating complex interactions between development changes and *U*_*max*_.

In adults, we were able to detect a significant difference in performance between male and female zebrafish, with adult males reaching maximal swimming speeds that were approximately 3 SL s^−1^ faster than females, regardless of age. Our results are very similar to the sex-related difference in zebrafish *U*_*crit*_ obtained from a large data set provided by Conradsen et al. [13]. Differences in morphology [24, 25], such as the greater girth of gravid females, and physiology, such as a reduced power output of muscles from gravid fish [26], have been proposed as mechanisms responsible for sex-related differences in fish swimming performance.

Finally, our flume and evaluation protocol was able to detect a 15–20% slower *U*_*max*_ in old compared to young adult fish. Few zebrafish survive longer than 1 year in the wild [27] but may live 3.5 to 5.5 years under laboratory conditions [28, 29]. Around 24 months of age, laboratory raised zebrafish experience an increased incidence of mortality associated with cachexia and spinal curvature [29]. Consistent with this early onset senescence, Gilbert et al. [11] reported that *U*_*max*_ was about 33% lower in 25–30 month old zebrafish compared to 8–12 month adult fish. Here, we show that as early as 22 months, *U*_*max*_ is reduced in both male and female zebrafish in comparison to younger adults, although the magnitude of decline is less than the older fish studied by Gilbert et al. Taken together, these data support the idea that reductions in the physical performance of laboratory-raised zebrafish can be detected around the mid-point of their lifespan.

Our flume and *U*_*max*_ protocol fulfills the need for a medium-throughput tool capable of assessing locomotion in zebrafish larvae, juveniles, and adults. Because the hardware and software are either off-the-shelf or open source, the flume is inexpensive, easily constructed, and readily adaptable. These features make it a useful tool for studying swimming performance of zebrafish in numerous academic disciplines, such as biomedicine, toxicology, environmental science, and ecology.

## Supporting information

S1 Appendix Flume components and assembly. Assembly of the flume and frame, including schematic figures and bill of materials.
(PDF)

S2 Appendix Electronics and software. Details regarding the microprocessor and electronic components, including a bill of materials, and an overview of the Arduino IDE and FlowControl software for programming the microcontroller.
(PDF)

S3 Appendix Calibration and operation. Instructions for calibrating the flow meters, establishing the PWM by flow rate relationship, and general principles for operating the flume.
(PDF)

S4 Appendix Data output. An example of data output by the microprocessor during flume operation.
(PDF)
