# Supplementary material for: An open source microcontroller based flume for evaluating swimming performance of larval, juvenile, and adult zebrafish
Source: PLoS One. 2018 Jun 26;13(6):e0199712. doi: 10.1371/journal.pone.0199712 (PMC6019105; doi:10.1371/journal.pone.0199712)
Supplement: S1 Appendix — Assembly of the flume and frame, including schematic figures and bill of materials. (PDF) [file pone.0199712.s001.pdf]

S1 Appendix: Flume components and assembly

Figure Fig S1-1 is an expanded view of the flume components and how they are assembled. A list of components and potential sources are compiled in Table S1-1. Short sections of 1 inch diameter polyvinyl chloride (PVC) tubing were used as couplers to join components. The use of these couplings allowed components to be easily disassembled for maintenance and cleaning while providing a degree of flexibility to the structure that was beneficial when loading fish into the working section. To prevent leaks, all threaded surfaces were wrapped with pipe thread sealant tape before assembly.

Modifications to components

The reservoir was made from a 7 l aquarium obtained from our zebrafish facility modified as follows: 1) a 1.875 inch hole was drilled in the bottom of the reservoir to accept the through hole pipe fitting, 2) two 0.75 inch holes were drilled in the upper sides of the reservoir as entry points for the return flow from the pumps. The polycarbonate tube working section was cut to length and a 0.25 inch hole was drilled through one wall of the tube near the downstream end as shown in Fig 1-B.

Pumps

Water circulation through the flume was provided by two industrial pumps that could be independently regulated via PWM. Each pump consisted of a magnetically driven spherical impeller that rotated at high speed on a ceramic bearing. The pumps should always contain water when operating as running them dry can result in irreversible damage to the bearing. The pumps were configured so that in the absence of PWM, they defaulted to their maximum speed. This results in an inverse relationship between the PWM signal and pump output. Be aware that for prolonged, sustained use at high outputs, the pumps will generate sufficient heat to alter the temperature of the flume water. We did not experience this problem with the relatively short duration protocols used in the present study.

Flow meters

Hall-effect flow meters were used to measure flow through the flume. We found these meters were highly linear with very little inter-meter variability (see Fig 3). In a preliminary design we used an infrared flow meter. This meter performed very well and is an acceptable substitution. However, we found that it was more sensitive to debris or waste that may be inadvertently circulating through the flume.

Frame

A frame for supporting the reservoir, working section, and pumps is illustrated in Fig S1-2 and a list of parts and potential suppliers are compiled in Table S1-2. The base was constructed out of T-

slotted aluminum framing held together with inside corner brackets. Common laboratory aluminum rod, bench supports, and connectors were used to form supports for the reservoir and the working section as shown. The pumps were bolted directly into the aluminum framing. Alternatively, investigators can use their ingenuity and miscellaneous materials to construct a serviceable supporting structure for the flume.

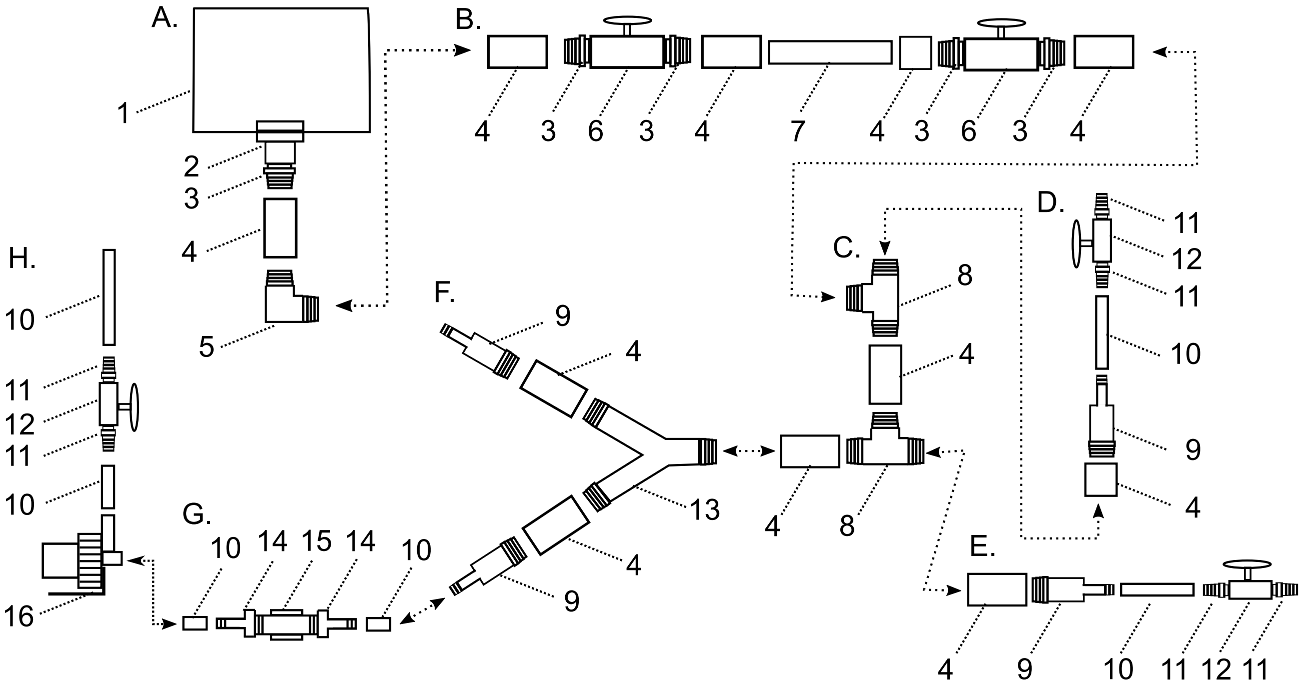

Figure S1-1. Expanded schematic diagram of the flume and assembly notes. A: Reservoir assembly. B: Working section assembly. C: Flow return assembly. D: Bubble trap assembly. E: Drain assembly. F: Fork assembly. G: Flow meter assembly (two assemblies required). H: Pump assembly (two assemblies required). Individual components were joined using lengths of 1 inch diameter PVC tubing (part no. 4). To prevent leaks, all threaded surfaces were wrapped with pipe thread sealant tape before assembly. See Table S1 for a list all of the components illustrated here.

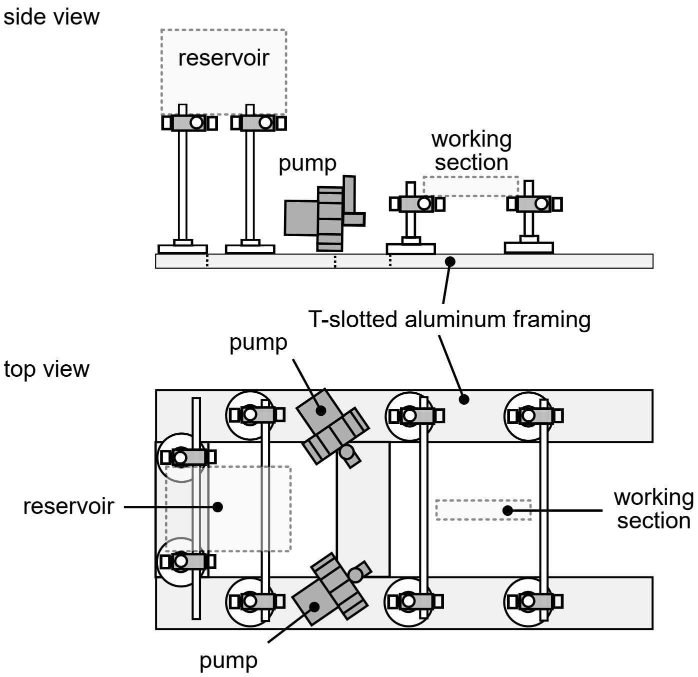

Figure S1-2. Schematic diagram of frame for holding flume. Parts listed in Table S2.

Table S1-1. Flume components

	component	source	part no.	quantity	cost ($)
1.	reservoir			1	
2.	through wall pipe fitting, 1 NPT	mcmaster.com	36895K163	1	24
3.	fitting, 1 NPT male x 1 in. barb	mcmaster.com	48315K93	5	7
4.	PVC tubing, 1 in. ID x 1.25 in. OD	mcmaster.com	5233K72	1 m	15[a]
5.	connector, 90° elbow, 1 in. barbs	mcmaster.com	48315K33	1	3
6.	PVC valve, 1 NPT female	mcmaster.com	976K13	2	32
7.	polycarbonite tube, 1 in. ID x 1.125 in. OD	mcmaster.com	8585K62	10 cm	19[a]
8.	connector, tee, 1 in. barbs	mcmaster.com	48315K63	2	7
9.	reducer, 1 in. barb x 0.5 in. barb	mcmaster.com	5463K653	4	10[b]
10.	PVC tubing, 0.5 in. ID x 0.625 OD	mcmaster.com	5231K371	1 m	7[a]
11.	connector, 0.5 male NPT x 0.5 in. barb	mcmaster.com	48315K91	8	8
12.	PVC valve, 0.5 female NPT	mcmaster.com	4876K11	4	31
13.	connector, Y, 1 in. barbs	usplastics.com	65355	1	3
14.	adapter, 0.5 female NPT x 0.5 in. barb	grainger.com	22FN26	4	10
15.	Hall-effect flow meter	adafruit.com	828	2	20
	or infrared flow meter	swissflow.com	SF800	2	120
16.	Swiftech model MCP655-PWM pump	frozencpu.com	ex-pmp-230	2	210
	stainless steel mesh disks, 1 in. diameter:				
	20 x 20 mesh	mcmaster.com	2930T13		4
	40 x 40 mesh	mcmaster.com	2930T33		4
	100 x 100 mesh	mcmaster.com	2930T53		4
	pipe thread sealant tape	mcmaster.com	6802K14		5

Numbers in left-most column refer to components labeled in Fig S1. [a] cost for 10 feet; [b] cost for 10 units. Abbreviations: NPT, National Pipe Taper; in., inch; PVC, polyvinylchloride; ID, inner diameter; OD, outer diameter.

Table S1-2. Frame components.

component	source	part no.	quantity	cost
T-slotted aluminum framing	8020.net			
framing, sides		25-2550	2 @ 75 cm ea.	21
framing, end and cross-piece		25-2550	2 @ 25 cm ea.	10
inside corner brackets		25-4119	6	18
bolts, M6 x 16 mm		13-6316	36	11
slide-in T-nuts, M6		25-1961	36	10
washers, M6		13-6041	36	8
aluminum rod, 1/2 inch	mcmaster.com	88615K13	6 feet	20
rod base	harvardapparatus.com	53-2302	8	72
closed connector	harvardapparatus.com	53-2012	8	80

Cost is product of unit cost times quantity, rounded to nearest dollar.
