# Supplementary material for: An open source microcontroller based flume for evaluating swimming performance of larval, juvenile, and adult zebrafish
Source: PLoS One. 2018 Jun 26;13(6):e0199712. doi: 10.1371/journal.pone.0199712 (PMC6019105; doi:10.1371/journal.pone.0199712)
Supplement: S2 Appendix — Details regarding the microprocessor and electronic components, including a bill of materials, and an overview of the Arduino IDE and FlowControl software for programming the microcontroller. (PDF) [file pone.0199712.s002.pdf]

S2 Appendix: Electronics and software

Microcontroller and IDE

A list of electronics parts and potential suppliers are compiled in Table S2-1. An Arduino model 101 microcontroller platform was used to control and monitor the flume. Other Arduino models, such as the Uno, may also be acceptable as would third-party boards based on the Arduino 101 or Uno designs. The board was programmed using the Arduino Independent Development Environment (IDE) which can be downloaded from the Arduino website (www.arduino.cc). The Intel Curie core and other drivers must be installed on the Arduino. Instructions and tutorials on downloading the IDE, configuring the controller, and using the Board Manager and the Library Manager can be found on the Arduino web site. The OneWire and the DallasTemperature libraries, available for installation using the Library Manager, must be installed for temperature measurements. The serial monitor, accessible through the IDE, is used to communicate with the Arduino and to display the current status of the flume. A tutorial on using the serial monitor can be found at the adafruit web site (https://learn.adafruit.com/lesson-0-getting-started/the-lessons).

FlowControl software

The current version of our flume control program, FlowControl, has been deposited in a repository on GitHub (https://github.com/jjwidrick/flume-project). FlowControl is released under the MIT license. A document detailing software set-up is included in the repository. Here, we provide an overview of the software.

The flume control program will open in the Arduino IDE. The beginning of the code is heavily commented. Investigators should scroll through these comments and refer to the software documentation in the GIT repository for the most up-to-date directions on configuring the program. Once this information is complete, the investigator can use the menu commands in the IDE to compile and upload the program to the microcontroller. Once the program has been uploaded, the investigator switches to the serial monitor and selects a mode of operation from the options presented.

Every 5 s, the program will update the options and display the water temperature. This allows investigators to bring the water to operating temperature before initiating calibration or data collection. The total time in mode 1 (a custom protocol) and mode 3 (the PWM verses flow calibration) are set by the investigator during configuration of the software. When mode 2 (manual control) is launched, the user is asked to enter the total minutes the flume runs in this mode.

To exit any current mode before time expires, the investigator throws the toggle switch to its opposite position. Flow will be shut-off at the start of the next data acquisition cycle, returning control to the options window.

Table S2-1. Electronic components.

component	source	part no.	quantity	cost ($)
Arduino microcontroller	arduino.cc	model Uno or 101	1	22-30
power adapter, 12V, 5A	jameco.com	2226038	2	42
USB-A male to USB-B male cable	jameco.com	222010	1	3
DS18B20 temperature sensor	sparkfun.com	SEN-11050	1	10
rocker switch	jameco.com	577221	3	6
potentiometer	jameco.com	29082	1	2
resistor, 10 kΩ	jameco.com	691104	1	< 1
resistor, 4.7 kΩ	jameco.com	661677	1	< 1
small breadboard	jameco.com	2109801	1	4
hook-up wire	jameco.com	2152876	1	4
