# Supplementary material for: An open source microcontroller based flume for evaluating swimming performance of larval, juvenile, and adult zebrafish
Source: PLoS One. 2018 Jun 26;13(6):e0199712. doi: 10.1371/journal.pone.0199712 (PMC6019105; doi:10.1371/journal.pone.0199712)
Supplement: S3 Appendix — Instructions for calibrating the flow meters, establishing the PWM by flow rate relationship, and general principles for operating the flume. (PDF) [file pone.0199712.s003.pdf]

S3 Appendix: Calibration and operation

The flow meters must be calibrated and the calibration constants entered into and saved in the flume control program. Once these values have been entered, the program is ready for data collection.

A typical data collection session consists of the following steps: running the PWM verses flow calibration protocol (mode 3), calculating and entering the PWM verses flow calibration constants into the flume control program, creating or recalling a test protocol, and using this protocol to control the flume.

We recommend draining the flume and dissembling major components after data collection on order to minimize bacterial growth in the flume. When the flume is next prepared for use (either flow meter calibration or data collection), it must be purged of any air trapped as the flume is filled with water.

Purging air

Filling the flume with water will inevitably trap air in some components. Any air drawn into the pumps will severely impair their output, may shortening their operating life, and has the potential to invalidate the PWM verses flow calibration. The following steps describe how to purge air from the flume.

1. The pumps should be powered off but with both outflow valves open.
2. Make sure the access port of the working section is closed with Parafilm and the bubble trap valve is closed. Then fill the flume by pouring ≈ 3.5 liters of fresh fish water into the reservoir.
3. Carefully peel back the Parafilm closure covering the port to purge air from the working section (tilting the working section slightly helps move air bubbles to the port) and then re-seal. Open the bubble trap valve, allow air to escape, and then re-close the valve.
4. Purge air from pump 1 as follows. Close the outflow value for pump 2. Go into mode 2 (manual mode), power up pump 1 only, and turn the potentiometer to yield maximum output. The maximum output of a single pump is ≈ 40 ml s^{-1}, but most likely there will be air trapped in the pump reducing its output. Turn off pump 1. In a few seconds, any air trapped in the pump 1 should rise through the outflow tubing. Re-start pump 1. It may require several stop/start cycles to purge all of the air that has been drawn into pump 1.
5. Power down pump 1, close its output valve and purge air from pump 2 in a similar manner.
6. Finally, run both pumps simultaneously by powering them up, selecting mode 2, manually setting output to maximum, and then opening both outflow valves. When all air is purged, the pumps should run very smoothly and quietly and attain a combined output of $70 - 74$ cm s^{-1}. It may require several iterations between purges of each pump to arrive at this point. However, once this is attained, the small amount of air trapped in the flume when adding and removing fish can usually be purged manually from the working section port and the bubble trap.
7. Note that when one pump is running and the second is powered off, it is necessary to keep the outflow valve of the second pump closed to prevent backflow through the pump.

Backflow will drain the pump (requiring a re-purging of the system) and confound the flow measurements. Likewise, when one pump is running and the second is then powered on, the outflow value to the second pump should remain closed for a few seconds in order to allow the second pump to build up positive water pressure.

Flow meter calibration

The goal is to collect water entering the reservoir for a well-defined period of time, determine its volume, calculate a flow rate, and then link this flow rate with the pulse frequency output by the flow meter during the water collection period. Following are step-by-step instructions for calibration.

1. Fill the flume with water, upload the flume control program, and purge air from the flume.
2. Select the flow meter for calibration. Power down the opposite pump and close its outflow valve.
3. Select mode 2 and use the potentiometer to dial in the first calibration flow rate.
4. Obtain a suitable container and collect water as it is ejected into the reservoir. We view the serial monitor to initiate and terminate collection over an appropriate multiple of 5 s. Measure and record the volume of water collected. The serial monitor also displays the flow meter pulse frequency (in Hz) every 5 s allowing one to calculate a mean frequency for the collection time period.
5. Repeat the above across the range of flow velocities expected. Use Fig 3 as an example.
6. To obtain slow flow rates, restrict pump output (and thus the volume of water drawn through the flow meter) by partially closing the outflow valve from the active pump.
7. Regress flow, in ml s^{-1}, against flow meter Hz and enter the slope and y-intercept as calibration constants into the flow control program.

PWM verses flow calibration

The goal is to determine the relationship between the PWM signal output by the microcontroller and the corresponding flow through the flume. The flume should be configured exactly as it will be used for data collection, i.e with an appropriate down- and upstream screens, etc. Assuming air has been purged, follow these steps.

1. Run the flume in mode 2 at maximum output for several minutes to warm-up the pumps and to bring water to the temperature used during data collection.
2. Run mode 3. The flume will automatically progress through a series of flow rates.
3. At the conclusion of the calibration run, copy results, and paste into a text file.
4. Calculate the average flow rate (in cm s^{-1}) over the final 15 s at each PWM level. Use a second order polynomial regression to obtain first, second, and y-intercept calibration coefficients for the relationship between PWM and flow rate. See Fig 4 for examples.
5. Exit the flume control program. Follow instructions in the comments of the program and enter calibration coefficients at the appropriate place in the source code.
6. Re-load the flume control program. You should now be ready to collect data, using either mode 1 (a custom protocol) or mode 2 (manual control)

7. For slow flow rates, run mode 3 with only one pump powered up and the outflow value from the pump partially closed. Note that during data collection, this valve must remain in the same partially closed position in which it was calibrated. If this valve is perturbed after calibration, then the relationship between PWM and flow rate has likely been altered and the previous calibration is no longer valid.
