# Supplementary material for: An open source microcontroller based flume for evaluating swimming performance of larval, juvenile, and adult zebrafish
Source: PLoS One. 2018 Jun 26;13(6):e0199712. doi: 10.1371/journal.pone.0199712 (PMC6019105; doi:10.1371/journal.pone.0199712)
Supplement: S4 Appendix — An example of data output by the microprocessor during flume operation. (PDF) [file pone.0199712.s004.pdf]

S4 Data output

An electronic record of the trial can be obtained by highlighting the results in the serial monitor (it is helpful to disable auto-scrolling when doing this) and copying the results to the clipboard. The results can then be pasted into a text editor and saved as a .txt file for a permanent record of the trial. Table S4-1 shows a small portion of a record copied from the serial monitor.

Table S4-1. Sample of data output.

date	ID	stage	sec	total	tempC	target	PWM1	PWM2	Hz1	Hz2	mls1	mls2	mls	cms
171010	3	5	5	130	25.1	20	208	208	20.75	17	51.42	42.01	93.43	18.45
171010	3	5	10	135	25.1	20	208	208	19.5	24	48.37	58.67	107.04	21.14
171010	3	5	15	140	25.1	20	208	208	18.5	22	45.93	53.91	99.84	19.71
171010	3	5	20	145	25.1	20	208	208	19.75	20.75	48.98	50.94	99.92	19.73
171010	3	5	25	150	25.1	20	208	208	17.5	22.5	43.49	55.1	98.59	19.47
171010	3	5	30	155	25.2	20	208	208	22.25	17.75	55.08	43.8	98.87	19.52
171010	3	6	5	160	25.2	22.5	202	202	20	24.25	49.59	59.27	108.86	21.49
171010	3	6	10	165	25.2	22.5	202	202	20.75	25	51.42	61.05	112.47	22.21
171010	3	6	15	170	25.2	22.5	202	202	20.75	25	51.42	61.05	112.47	22.21
171010	3	6	20	175	25.2	22.5	202	202	18.25	26.25	45.32	64.03	109.35	21.59
171010	3	6	25	180	25.2	22.5	202	202	23.5	25.5	58.13	62.24	120.37	23.77
171010	3	6	30	185	25.2	22.5	202	202	23.25	23.75	57.52	58.08	115.6	22.82

Abbreviations: ID, subject identification; sec, seconds in current stage; total, total seconds since start of protocol; tempC, temperature in °C; target, target flow rate in cm s^{-1}; PWM1 and PWM2, pulse width modulation value for pump 1 and pump 2, respectively; Hz1 and Hz2, pulse per s output of flow meter 1 and flow meter 2, respectively; mls1 and mls2, flow rate in ml s^{-1} through flow meter 1 and flow meter 2, respectively; mls, total flow rate in ml s^{-1}; cms, total flow rate in cm s^{-1}.
